# Imaging Features of Intratumoral Injection of NBTXR3 for Head and Neck Squamous Cell Carcinoma Lymph Node Metastases

**DOI:** 10.3390/diagnostics12092156

**Published:** 2022-09-05

**Authors:** Daniel Thomas Ginat, Aditya Juloori, Omar I. Vivar, Leonard A. Farber, Zhen Gooi, Ari J. Rosenberg

**Affiliations:** 1 Department of Radiology, University of Chicago Medical Center, Chicago, IL 60637, USA; 2 Department of Medicine, University of Chicago Medical Center, Chicago, IL 60637, USA; 3 Department of Global Medical Affairs, Nanobiotix, 75012 Paris, France; 4 Department of Surgery, University of Chicago Medical Center, Chicago, IL 60637, USA

**Keywords:** NBTXR3, injection, tumor, head and neck, cancer

## Abstract

NBTXR3 nanoparticle injection is a relatively novel radioenhancer for treatment of various cancers. CT scans following NBTXR3 injection of metastatic lymph nodes from head and neck squamous cell carcinoma were reviewed in a small series of patients. The radioenhancing appears as hyperattenuating, with a mean attenuation of the injected material of 1516 HU. The material was found to leak beyond the margins of the tumor in some cases.

## 1. Introduction

NBTXR3 is a first-in-class radioenhancer composed of crystalline hafnium oxide nanoparticles functionalized by a negatively charged surface coating with a size centered on 50 nm. The nanoparticles augment the absorption of ionizing radiation, resulting in increased tumor cell death. Intratumoral injection of NBTXR3 followed by radiation therapy has been found to be feasible and safe for a variety of tumors, such as locally advanced squamous cell carcinoma of the oral cavity or oropharynx and soft tissue sarcomas [1,2,3]. However, there is a paucity of reports describing the imaging findings of NBTXR3 injection for the treatment of patients with head and neck squamous cell carcinomas. Thus, the purpose of the study is to characterize the CT imaging features of the injected NBTXR3 in head and neck squamous cell carcinoma lymph node metastases.

## 2. Methods

This retrospective imaging review was performed under a prospective trial approved by the institutional review board. The patients included in this study had a diagnosis of head and neck squamous cell carcinoma that was recurrent in a previously irradiated field with limited therapeutic options. The patients were enrolled in a clinical trial of radiation therapy to the injected lymph node followed by initiation of anti-PD1 immunotherapy after completion of radiation (NCT03589339). NBTXR3 was supplied by Nanobiotix S.A. as a sterile aqueous suspension of nanoparticles composed of functionalized hafnium oxide crystallites. The injection volume of NBTXR3 was pre-specified per protocol and was determined by volume of the target lesion as assessed on diagnostic imaging and the injection was performed under ultrasound guidance. The initial soft tissue neck CT scans (3 mm sections, 120 KVP, 100 mAs) obtained following NBTXR3 injection were reviewed by a board-certified head and neck radiologist with about 10 years of experience for the distribution of the injected material within or adjacent to the lesion. The extent of injected material with respect to the tumor volume was estimated visually based on the CT scans and expressed in quartile ranges. In addition, the attenuation of the injected material was measured using manually drawn circular regions of interest on two representative slices and reported as the mean of the two measurements.

## 3. Results

Four patients were identified that underwent NBTXR3 injection of metastatic lymph nodes from head and neck squamous cell carcinoma with available post-injection CT scans (Table 1). The CT scans were obtained between 1 and 18 days following injection and the imaging findings are summarized in Table 2. The mean attenuation of the injected material in all four cases was 1516 HU. The material was present to variable degrees within the tumor, ranging from the lowest to highest quartiles. In addition, there was injected material beyond the margins of the tumor, or leakage, in three cases.

## 4. Discussion

This study shows that the injected NBTXR3 nanoparticle solutions are readily identifiable on CT at least up to several weeks following the injection. The material appears as markedly hyperattenuating deposits without significant beam-hardening artifact, which facilitates delineating the dispersion (coverage of the tumor volume) of the injected material. The hyperattenuating appearance is attributable to the hafnium oxide component, which has the desirable property of high X-ray attenuation for treatment purposes as a radioenhancer [1]. This property is also useful for verifying the dispersion of the material with respect to the target tumor. The radioenhancer is much more hyperattenuating than the typical contrast enhancement of lymph nodes. Otherwise, the radioenhancer material could potentially mimic dystrophic calcifications on CT, but the appearance of the substance would coincide with the timing of the injection and comparison with pre-injection scans could be useful for excluding this possibility.

Based on this series, the injected material typically displays irregular margins due to how it permeates the tissues. The presence of the radioenhancer material beyond the margins of the tumor is attributable to leakage from the tumor, presumably due to elevated pressures from the injection, which results in infiltration of the adjacent fat planes. Furthermore, the consistency of the tumor could impact the dispersion of the injected material. For example, the solid lesion demonstrated the most prominent leakage of the injected nanoparticles as compared to the necrotic tumors. Thus, this characteristic of the tumor should be considered when injecting the nanoparticles. It is possible that other features of the injected tumor, such as size and extracapsular spread, can lead to variations in the dispersion of NBTXR3, but this can be further studied via a larger cohort. It is also conceivable that the injected radioenhancers could spread through the regional lymphatic channels. Previous clinical studies have not commented on the presence of injected material beyond the tumor margins consistent with the reported safety profile. The presence of injected material beyond the tumor margins observed in this report did not have a negative impact on the safety or toxicity of the normal surrounding tissues. It has been suggested that as little as 10% of tumor volume injection in conjunction with radiotherapy is adequate for patients with soft tissue sarcomas [3].

It is suspected that the attenuation of the injected nanoparticle solution remains elevated for a prolonged period given that the case imaged at 18 days after injection displayed similar Hounsfield units to the other cases imaged immediately after injection. However, longer-term studies can address the longevity of the imaging results for the nanoparticles. Further studies to assess the significance of variations in degree of tumor volume injected and dispersion of the injected material in terms of efficacy and complications for head and neck squamous cell carcinoma are currently ongoing.

## 5. Conclusions

Intralesional NBTXR3 nanoparticle injections used as radioenhancers for the treatment of head and neck cancer lymph node metastases can be readily delineated on CT scans as hyperattenuating material. This property helps verify adequate delivery of the radioenhancers for subsequent therapy.

## Figures and Tables

**Table 1:** Patient characteristics.

Case	Age	Gender	Primary Cancer Site
1	70	Male	Tonsil
2	51	Male	Larynx
3	67	Female	Larynx
4	67	Female	Hypopharynx

**Table 2:** Imaging findings.

Figure	Quartile of Tumor Volume Injected (%)	Attenuation (HU)	Pre-Injection Imaging	Post-Injection CT
1	75–100	860	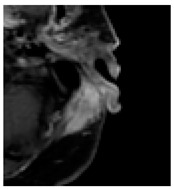 Axial post-contrast fat-suppressed T1-weighted MRI shows a solidly enhancing left retroarticular tumor.	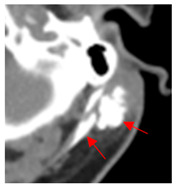 Axial CT image obtained 1 day after injection shows the NBTXR3 nanoparticles (arrows) in the tumor with leakage into the surrounding soft tissues.
2	0–25	1564	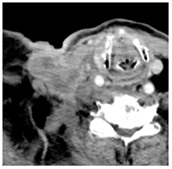 Axial CT image shows a partly necrotic tumor in the right neck.	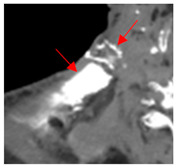 Axial CT image obtained 18 days after injection shows the NBTXR3 nanoparticles (arrows) in the tumor.
3	25–50	1716	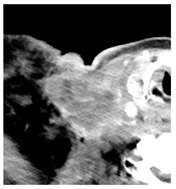 Axial CT image shows a mostly necrotic tumor in the right neck.	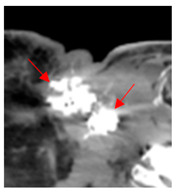 Axial CT image obtained 1 day after injection shows the NBTXR3 nanoparticles (arrows) in the tumor with a small amount of leakage into the surrounding soft tissues.
4	0–25	1923	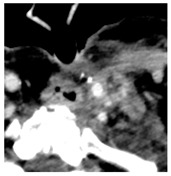 Axial CT image shows a mostly necrotic tumor in the left neck.	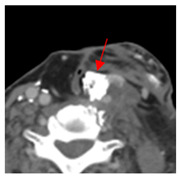 Axial CT image obtained 1 day after injection shows the NBTXR3 nanoparticles (arrows) in the tumor.

## Data Availability

Not applicable.
